# Autophagy: The Last Antioxidant to Unravel

**DOI:** 10.3390/antiox14101244

**Published:** 2025-10-17

**Authors:** Álvaro F. Fernández, Marina García-Macia

**Affiliations:** 1 Departamento de Bioquímica y Biología Molecular, Universidad de Oviedo, 33006 Oviedo, Spain; 2 Instituto Universitario de Oncología del Principado de Asturias (IUOPA), 33007 Oviedo, Spain; 3 Instituto de Investigación Sanitaria del Principado de Asturias (ISPA), 33011 Oviedo, Spain; 4 Institute of Functional Biology and Genomics (IBFG), Universidad de Salamanca-CSIC, 37007 Salamanca, Spain; 5 Department of Biochemistry and Molecular Biology, Universidad de Salamanca, 37007 Salamanca, Spain

Christian de Duve used the term “Autophagy” for the first time during a conference focused on lysosomes in 1963, but the scientific revolution caused by this cellular recycling system could not be foreseen at that moment. More than fifty years later, Yoshinori Oshumi was awarded the Nobel Prize for the description of the autophagy-related (ATG) genes, and to this very date, key aspects of this cellular process have been reported by hundreds of groups all over the world. Autophagy is a fundamental cellular process that maintains homeostasis, as it eliminates undesirable cytoplasmic components. It can be activated in response to various stresses, such as nutrient deprivation, hypoxia, drugs, and infections. Recent strong evidence indicates that autophagy is a crucial mediator in the regulation of the oxidative stress response. The more obvious role, when encountering oxidative stress, is removing oxidized proteins or organelles, including damaged mitochondria that generate excessive ROS. However, the knowledge about the interplay between autophagy and the antioxidant transcriptional factor Nrf2 opened the door to a myriad of new roles of autophagy as an antioxidant process. Exploring when and how autophagy modulates antioxidant defenses seems completely necessary. For this reason, we set up this Special Issue, aiming to provide a comprehensive platform for researchers to delve into the cross-regulation between autophagy and oxidative stress.

In this regard, we have included interesting reviews about the mechanistic aspects of the autophagy and oxidative stress interplay (Contributions 1 and 2), a crosstalk that could lead to new forms of cellular death (Contribution 3). Unraveling the basic mechanisms that control this interplay is the cornerstone for further understanding its role in different diseases. Additionally, this Special Issue also displays manuscripts focusing on how the antioxidant stimulation of autophagy is triggered upon certain pathological conditions (Contributions 4 to 9). We did not only focus on human health; very interesting articles about how autophagy can be a tool to improve animal welfare were also included in this Special Issue (Contributions 10 and 11).

Cells try to maintain homeostasis through different pathways that can be activated at the same time or in a sequential order. One of these signaling pathways is the route of the mitogen-activated protein kinases (MAPKs), so it is not a surprise that MAPKs, activated by ROS, are able to modulate autophagy, as it is deeply explained in Contribution 1. The source of ROS is also discussed in this Special Issue. While the main origin of ROS is mitochondria, we often neglect to acknowledge the importance of peroxisomes. Strikingly, peroxisomes also have antioxidant enzymes, so they can be central to the regulation of oxidative stress. When peroxisomes are not functional, they are recycled by a specific type of autophagy: pexophagy. This nexus between peroxisome-dependent oxidative stress regulation and the role of pexophagy is reviewed in Contribution 2. Moreover, these authors go further, addressing the relevance of pexophagy for neurodegenerative diseases too. Interestingly, even though autophagy is mostly considered a pro-survival mechanism, it can be aberrantly activated and lead to cellular death. Autosis is one of these types of death, and it is described thoroughly in Contribution 3. Defects in redox balance and accumulation of oxidative damage led to cellular death too, but the actual role of ROS in autosis is not well understood. The authors of this contribution review what is known about the interplay of ROS in autosis and suggest some audacious hypotheses to test in the future.

Aging is a complex process where both autophagy and oxidative stress participate. Autophagy generally declines with age while oxidative stress builds up. Also, senescent cells accumulate with aging, which can be established as a mark of a more detrimental decay. The use of different types of antioxidants is one of the recent strategies to improve aging and prolong lifespan (Contributions 4 and 5). These authors used flavonoid-related molecules that can be found in grapes, such as procyanidin A1 from peanut skin extract (PSE) and delphinidin, which impact the upstream steps of the autophagic pathway. In these articles, authors blocked autophagy at different levels and found that the antioxidant effect was eliminated. A different strategy, a probiotic yeast (Milmed), was used in Contribution 6. In this case, the beneficial effects of this antioxidant were also tested in vivo, and Milmed increased lifespan in C. elegans.

Aging is the most important risk factor for many diseases, such as cancer. Autophagy and oxidative stress also play a crucial role during the development of this pathology. Tumoral hypoxia or nutrient deprivation fosters oxidative stress that, in turn, activates autophagy to promote tumor cell survival. This is studied deeply in Contribution 7. Additionally, neurodegeneration and alcohol-related brain damage were addressed in Contribution 8. The authors establish the activation of CYP2E1 as the difference between both disorders, while autophagy and oxidative stress aspects are very similar in both diseases. In terms of therapies, stem cells and regenerative medicine are the focus of Contribution 9. Stem cell biology requires both autophagy and oxidative stress in fine balance since they contribute to cell survival, self-renewal, and differentiation. The fate of stem cells is critical to address age-related decline and enhance the potential of regenerative therapies.

Several reasons justify animal welfare research, but the economic value outweighs many of them. In this Special Issue, we learn how pollution affects chicken kidneys and produces nephrotoxicity, increasing oxidative stress and reducing autophagy. This problem severely impacts egg and meat production. Here, authors from contribution 10 tackle the problem with a strategy that is similar to the one previously mentioned in papers focusing on aging. This time, using another flavonoid, luteolin, in the diseased chickens. This treatment reduced oxidative stress and increased autophagy via SIRT1 activation, which resulted in the alleviation of the kidney damage. Apart from poultry, beef cattle are central to the livestock economy, mainly for milk production. Mastitis is a common illness in cows that reduces dairy production. Last but not least, Contribution 11 evaluates how *Streptococcus* infects mammary glands to produce mastitis. They discovered that mammary cells stimulated the antioxidant defenses through Nfr2 and Sirt1, and both activated autophagy.

In summary, this Special Issue provides new knowledge about the role of autophagy as a defense against oxidative stress. The numerous manuscripts cover everything from basic mechanisms to pathophysiology, not only related to human diseases but also considering animal welfare. But still, autophagy will for sure keep surprising the field with unexpected, new roles, as an antioxidant response, and more.

